# The role of physical acgtivity in life satisfaction and psychological resilience among college students: Mediating effects of self-efficacy and perceived stress

**DOI:** 10.1371/journal.pone.0331463

**Published:** 2025-09-02

**Authors:** Rujiang Zhang

**Affiliations:** College of Education and Sports Sciences, Yangtze University, Jingzhou, Hubei, China; Universitas Islam Negeri Raden Intan Lampung, HUNGARY

## Abstract

**Background:**

With the increasing global attention on mental health issues, especially the psychological stress and life satisfaction problems faced by college students, it has become particularly important to explore how physical activity is associated with college students’ psychological resilience and quality of life through psychological mechanisms. This study aims to examine the association between physical activity on college students’ life satisfaction and psychological resilience, and to investigate the mediating roles of self-efficacy and perceived stress.

**Methods:**

This study collected data from college students in several universities in China through online questionnaires, using the Body Self-Concept Questionnaire, Life Satisfaction Scale, the Chinese Revised Version of the Connor–Davidson Resilience Scale, General Self-Efficacy Scale, and Perceived Stress Scale to measure each variable. A total of 560 undergraduate students from three universities participated in the survey, reporting on their physical activity, life satisfaction, psychological resilience, self-efficacy, and perceived stress.

**Results:**

The findings show that physical activity was significantly associated with higher life satisfaction (*r *= 0.439, *p* < 0.001) and psychological resilience (*r *= 0.521, *p *< 0.001). Both self-efficacy (95% CI = [0.138, 0.255] and [0.245, 0.399]) and perceived stress (95% CI = [0.013, 0.070] and [0.040, 0.134]) played significant mediating roles in these processes. Specifically, physical activity was linked to better mental health and quality of life through its association with higher self-efficacy and lower perceived stress.

**Discussion and implications:**

This study validates the association between physical activity and improved college students’ life satisfaction and psychological resilience and reveals the mediating mechanisms of self-efficacy and perceived stress. These findings provide a scientific basis for designing exercise and psychological intervention programs for college students, with important practical implications.

## 1. Introduction

In today’s society, mental health issues have become one of the significant factors impacting people’s quality of life, particularly pronounced among college students. College students face various challenges, such as adapting to changing learning environments, coping with academic pressures, establishing interpersonal relationships, and planning future careers [1,2]. These challenges can affect not only their life satisfaction (LS) but also their psychological resilience (PR) adversely [3,4]. Furthermore, the levels of LS and PR can in turn directly influence college students’ academic performance, mental health, and the quality of interpersonal relationships [5,6]. The college phase is a critical period for cognitive development, where students’ assessments of their life conditions serve as vital indicators of personal well-being [7]. Having good LS and robust PR helps students improve their academic performance and promote holistic health development [8]. Therefore, exploring the factors influencing LS and PR among Chinese college students holds significant research and practical importance.

Previous studies have confirmed that an individual’s LS and PR are significantly influenced not only by personality traits, such as neuroticism [9], but also by external social factors (such as academic stress [10]) and behavioral habits (like physical activity (PA) [11]). PA, as a natural therapy requiring no medicinal intervention, offers a safe and effective means to enhance LS and PR among college students. Regular participation in PA naturally elevates their LS and PR without the side effects of medication [7,12]. Such activities stimulate the release of natural chemicals like endorphins, which not only alleviate pain but also enhance pleasure, thus directly boosting mood and LS [13]. Additionally, PA strengthens self-efficacy (SE), enhancing students’ confidence and ability to cope with daily stresses and challenges [14]. Furthermore, by improving sleep quality and increasing physical strength, PA further enhances the physiological and PR of college students, laying a solid foundation for their stable performance in academics and personal life [11,15]. Thus, PA is not merely a therapeutic measure, it is also a critical activity for prevention and promoting comprehensive health.

Despite the widely recognized positive effects of PA on mental health, research on its specific mechanisms of action remains relatively scarce. Particularly, the roles of SE and perceived stress (PS) as potential mediators in the impact of PA on mental health have not been thoroughly investigated. Although existing literature has explored the direct relationship between PA and mental health, or sporadically examined single mediators such as SE or PS, there is a lack of studies that systematically investigate the dual mediating roles of SE and PS in the relationship between PA, LS, and PR among college students. This study innovatively introduces both SE and PS as core mediating variables, aiming to explore their mediating roles in the process linking PA with LS and PR in college students, and to construct and validate a more comprehensive model of these pathways. This research design goes beyond the traditional focus on direct effects or single mediators, aiming to reveal a more complex psychological mechanism chain through which PA affects mental health. This research direction not only deepens our understanding of the psychological benefits of PA—particularly its dual pathways of promoting well-being by enhancing SE and alleviating stress perceptions—but also provides a new perspective and a solid theoretical foundation for designing more targeted and integrated mental health interventions.

The study aims to delve into how PA enhances college students’ LS and PR by affecting their SE and PS. By analyzing the mechanisms of these two mediators in detail, this study seeks to reveal the specific pathways through which PA impacts the mental health of college students. Furthermore, by providing empirical support, this study aims to offer a theoretical basis for universities and policymakers to design and implement effective physical intervention measures. We anticipate that these research findings will promote the overall health of college students, helping them achieve higher satisfaction and stronger PR in their academic and personal lives.

## 2. Literature review and research hypotheses

### 2.1. Physical activity and life satisfaction

PA refer to various forms of activities that involve physical movement and skills, aimed at improving physical health and fitness. These can include organized competitive sports, fitness training, yoga, running, and more [16]. LS is a crucial indicator that measures an individual’s contentment with their quality of life, encompassing both psychological state and environmental conditions [7,17]. Numerous studies have extensively explored the positive impact of PA on individual LS, finding that college students who regularly engage in physical activities often report higher LS [7,18,19]. For example, a study by Liu, Zhu and Jiang [19] demonstrates a strong positive correlation between regular participation in PA and higher LS among Chinese college students. PA enhances individual life quality directly and indirectly by improving physical health, strengthening social connections, and boosting psychological health [20]. Additionally, physiological changes induced by physical activities, such as the release of endorphins, also contribute to enhanced mental health, thereby increasing LS [13]. According to Self-Determination Theory (SDT), individuals’ participation in physical activities can fulfill the three basic psychological needs of autonomy, competence, and relatedness, thereby enhancing their LS [21]. When college students experience improvements in personal ability and social interaction through physical activities, their LS is significantly increased. Based on the above analysis, this study proposes the following hypothesis:

H1: PA significantly positively affects college students’ LS.

### 2.2. Physical activity and psychological resilience

PR refers to an individual’s capacity to proactively self-regulate and adapt positively to the environment when facing setbacks or adversity, in order to cope with complex and changing challenges [22]. Studies have shown that PA significantly enhances an individual’s PR [23,24]. SDT posits that the opportunities for autonomous choice and skill mastery provided by physical activities can enhance the fulfillment of psychological needs, which is the core mechanism of resilience development [21]. In several cross-cultural studies, individuals who regularly engage in PA demonstrate higher stress resistance and better emotional regulation skills [11,15]. For instance, research by Zhao, Zhao, Wang, Zhang and Chen [11] on Chinese college students found that PA significantly and positively enhances their PR. PA provides an effective stress-release mechanism, not only reducing tension and anxiety through physical activity but also improving mental states by enhancing feelings of achievement and self-control [12]. Additionally, PA strengthens social support networks, which play a crucial role in enhancing PR [25]. Based on the above analysis, this study proposes the following hypothesis:

H2: PA significantly positively affects college students’ PR.

### 2.3. The mediating role of self-efficacy

SE refers to an individual’s belief in their own abilities to successfully execute actions necessary to achieve specific goals [26]. According to Self-Efficacy Theory (SET), an individual’s PR and LS largely depend on their belief in their own abilities and self-regulation skills. PA as a positive mastery experience, not only enhances an individual’s SE but also effectively improves their ability to cope with adversity [27,28]. Research indicates that PA enhances LS and PR indirectly by boosting SE. Specifically, engaging in PA has been found to significantly increase SE [14,29], which in turn significantly enhances individuals’ LS [30,31]. For example, a study by Wang, Li, Zhang and Luo [14] explored the relationship between PA and SE among college students, finding a significant positive correlation. Research by van Zyl and Dhurup [30] also demonstrated that college students’ SE significantly predicted their LS. Further, a study found that by increasing the frequency of weekly exercise, participants’ exercise SE was enhanced, which in turn led to an increase in their LS [32]. This improvement is primarily realized through physiological and psychological positive changes brought about by exercise, such as improved cardiovascular health, increased physical strength, and enhanced emotional states [33,34].

According to SET, PA, as a mastery experience, can significantly enhance an individual’s SE [35,36]. When individuals make progress in physical fitness and skills through PA, this success experience directly boosts their SE, thereby increasing their confidence in facing other life challenges [14]. This enhanced SE not only enables individuals to cope more actively with stress but also strengthens their PR [37,38]. For example, research by Li, Wang, Yu, Liu, Xu, Lin, et al. [39] on the PR of adolescents showed that PA significantly enhanced individual PR, and SE played a mediating role in the relationship between PA and PR. Based on the above analysis, this study hypothesizes:

H3: SE mediates the relationship between college students’ PA and LS.

H4: SE mediates the relationship between college students’ PA and PR.

### 2.4. The mediating role of perceived stress

PS refers to an individual’s perception and evaluation of stressors in life, encompassing sensitivity to stress, ongoing perception of stress, and subjective interpretation of stress situations [40]. According to SDT, PS reflects an individual’s perception of pressure and their coping ability when facing external challenges [21]. PA is often regarded as an effective strategy for alleviating stress. For instance, Zhai, Wu, Koriyama, Wang, Shi, Huang, et al. [41] investigated the relationship between Chinese college students’ participation in physical activities and PS, finding that those who regularly engage in PA generally exhibit lower levels of PS. PA as an activity that helps alleviate stress, can indirectly enhance an individual’s LS and PR by reducing PS. Therefore, in the relationship between PA and LS, PS plays a key mediating role. Meyer, Grob and Gerber [42] studied the relationship between PA and LS among adolescents, discovering that PA not only enhances LS but also influences it by reducing PS. Additionally, other studies have indicated that regular physical activity can significantly reduce the perception of academic stress, thereby improving LS [43]. Thus, PA indirectly enhances LS by reducing PS, highlighting the importance of PS in psychological regulation [44].

The relationship between PS and PR profoundly affects how individuals cope with daily life stresses and challenges. Research shows that individuals who can effectively manage and reduce PS recover more quickly from major life events and demonstrate greater adaptability and resilience [45]. Lara-Cabrera, Betancort, Muñoz-Rubilar, Rodríguez Novo and De las Cuevas [46] explored the relationship between PS, PR, and mental health among nurses, finding that lower PS can enhance individual PR and promote mental health. In the relationship between PA and PR, PS plays a key mediating role. For example, a study found that college students participating in team sports not only reported lower PS but also experienced significant improvements in various aspects of PR—including emotional regulation, adversity coping, and resilience [47]. Therefore, reducing PS can be seen as an important mediating path through which PA positively influences PR, emphasizing the significance of physical activities in promoting mental health and adaptability. Based on the above analysis, this study hypothesizes:

H5: PS mediates the relationship between college students’ PA and LS.

H6: PS mediates the relationship between college students’ PA and PR.

**Fig 1:**
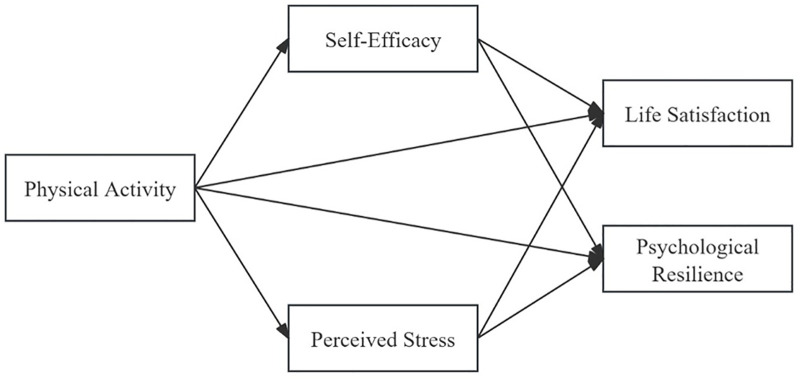
Research framework.

Based on the above assumption, the research framework is illustrated in Fig 1.

## 3. Materials and methods

### 3.1. Participants

From December 2024 to February 2025, this study collected data through the online questionnaire platform Questionnaire Star (www.Sojump.com) by randomly selecting participants from multiple universities in China. Initially, we established preliminary communication with the school administration and coordinated with counselors, who introduced the purpose and significance of the survey to students during class meetings. Next, participants were randomly selected from the student lists of each class using computer-generated random numbers, ensuring the randomness and representativeness of the sample. After the participants were selected, counselors shared the survey link in the class group and emphasized the voluntary nature of participation and privacy protection measures. This approach respected students’ willingness while ensuring the quality and reliability of the data, providing a solid foundation for subsequent analysis. This study was approved by the Ethics Committee of Yangtze University (Approval Number: No. 2024-010-014). All participants provided informed consent prior to the survey. For participants under the age of 18, written consent was obtained from their guardians.

According to Kline [48], the sample size should have at least 10 respondents per survey item. The questionnaire in this study included 59 items. Considering an approximate 20% sample attrition rate, the required sample size was determined to be 708 participants (102 items × 10 respondents + 20% × 102 items × 10 respondents). To meet the research requirements, 720 questionnaires were distributed, and 710 were eventually returned [49]. During the data cleaning process, 150 questionnaires were discarded for the following reasons: more than 20% of the questions were left unanswered, or more than 80% of the questions were answered with extreme options (completely agree or completely disagree). These response patterns could lead to significant bias, known as floor or ceiling effects, which would affect the accuracy of the data analysis. Therefore, the study analyzed data from 560 valid questionnaires.

The specific demographic data is shown in Table 1: 140 males (25.0%) and 420 females (75.0%); the majority of participants were between 19 and 22 years old, with 518 participants (92.5%); the largest proportion was second-year students, with 290 participants (51.8%). There were significant differences in LS and PR based on gender (*p* < 0.001) and grade level (*p* < 0.001). This representative sample provides a solid foundation for analyzing the current status of college students’ LS and PR. The diversity and scale of the sample contribute to ensuring the generalizability of the study’s findings to a broader college student population within this research context. All participants voluntarily participated in the study and signed a written informed consent form. The research process adhered to the ethical principles of the Declaration of Helsinki.

**Table 1:** Demographic characteristics of participants.

Variable	N	Percentage (%)	LS *p*-value	PR *p*-value
Gender			< 0.001	< 0.001
Male	140	25.0		
Female	420	75.0		
Grade			< 0.001	< 0.001
Freshman	216	28.6		
Sophomore	290	51.8		
Junior	52	9.3		
Senior	2	0.4		
Age			0.292	0.193
Under 18 years old	36	6.4		
19-22 years old	518	92.5		
Over 22 years old	6	1.1		

### 3.2. Measurement instruments

#### 3.2.1. Physical activity scale.

The PA scale in this study is derived from the Physical Self-Description Questionnaire (PSDQ) developed by Marsh, Richards, Johnson, Roche and Tremayne [50], specifically from the PA dimension. This dimension consists of six items, two of which require reverse scoring. The scale is typically rated on a 5-point scale, ranging from 1 (strongly disagree) to 5 (strongly agree). Higher scores indicate a higher level of PA among university students. In this study, the Cronbach’s α for the scale is 0.879.

#### 3.2.2. Life satisfaction scale.

The LS scale used in this study is the Satisfaction with Life Scale developed by Diener, Emmons, Larsen and Griffin [51], which assesses university students’ overall LS. This scale contains five items and utilizes a 7-point Likert scale, ranging from 1 (strongly disagree) to 7 (strongly agree). Higher scores reflect a higher level of LS. The scale has been validated among Chinese university students and has shown good reliability and validity [35]. In this study, the Cronbach’s α for the scale is 0.876.

#### 3.2.3. Psychological Resilience Scale.

The Chinese revised version of the Connor-Davidson Resilience Scale (CD-RISC), developed by Yu and Zhang [52], is used to assess university students’ PR. The scale consists of three dimensions—toughness, self-reliance, and optimism—and contains 25 items in total. Each item is rated on a 5-point scale, ranging from 1 (strongly disagree) to 5 (strongly agree). Higher scores indicate stronger PR. The scale has been validated in Chinese university student populations and has demonstrated good reliability and validity [53]. In this study, the Cronbach’s α for the scale is 0.976.

#### 3.2.4. General self-efficacy scale.

The General Self-Efficacy Scale [54] is used to measure university students’ SE in this study. The scale includes 10 items, each rated on a 5-point scale ranging from 1 (strongly disagree) to 5 (strongly agree). The total score is the sum of the individual item scores, ranging from 10 to 50. A higher total score indicates stronger general SE. This scale has been validated among Chinese university students and has shown good reliability and validity [55]. In this study, the Cronbach’s α for the scale is 0.948.

#### 3.2.5. Perceived stress scale.

The Perceived Stress Scale (PSS) developed by Cohen, Kamarck and Mermelstein [40] is used to measure participants’ PS levels. The scale consists of 10 items, each rated on a 5-point scale, ranging from 1 (strongly disagree) to 5 (strongly agree). The total score is the sum of the individual item scores, ranging from 10 to 50. Higher scores indicate higher PS levels. The scale has been validated among Chinese university students and has demonstrated good reliability and validity [56]. In this study, the Cronbach’s α for the scale is 0.872.

### 3.3. Statistical analysis

This study conducted data analysis using SPSS 22.0. To test the correlations between variables (H1 and H2), descriptive statistics and Pearson product-moment correlation analysis were employed. To examine the mediating roles of SE and PS (H3 to H6), Model 4 within the PROCESS macro in SPSS 22.0 was utilized. The study performed bootstrapping with 5000 samples to determine the mediation effects. If the bias-corrected Bootstrap 95% confidence interval (CI) does not include zero, it indicates a significant mediating effect at the α = 0.05 level.

## 4. Results

### 4.1. Test for common method bias

This study employed Harman’s single-factor test to assess Common Method Bias (CMB). The test revealed that no single factor accounted for a majority of the variance [57]. The largest single factor identified explained only 43.631% of the variance, significantly below the critical threshold of 50% [58]. Therefore, it can be concluded that common method bias does not significantly impact the results of this study.

### 4.2. Correlation analysis

The study conducted a correlation analysis on the total scores of PA, LS, PR, SE and PS. As shown in Table 2, Pearson product-moment correlation analysis indicated significant positive correlations between PA and LS, PR, and SE (*p* < 0.001). There was a significant negative correlation between PA and the total score of PS (*p* < 0.001). Thus, H1 and H2 are supported by these findings.

**Table 2:** Means, standard deviations, and correlation analysis of variables (*r*).

Variable	M ± SD	PA	LS	PR	SE	PS
PA	22.82 ± 4.12	1				
LS	24.41 ± 6.39	0.439***	1			
PR	95.43 ± 17.54	0.521***	0.678***	1		
SE	37.22 ± 7.28	0.433***	0.590***	0.877***	1	
PS	25.92 ± 6.97	−0.255***	−0.289***	−0.533***	−0.606***	1

Note: N = 560. PA, physical activity; LS, life satisfaction; PR, psychological resilience; SE, self-efficacy; PS, perceived stress; ***p < 0.001.

### 4.3. Analysis of mediating effects

#### 4.3.1. The mediating role of self-efficacy.

**Fig 2:**
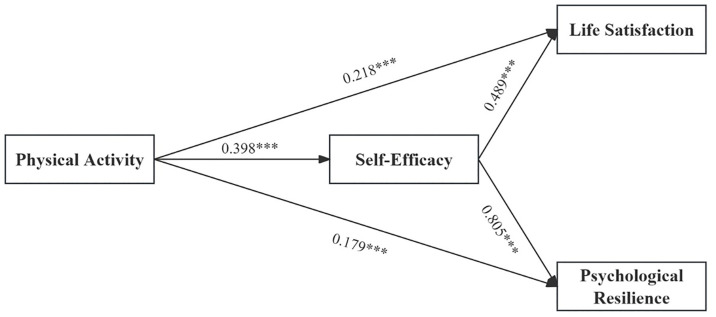
Results of the mediating effect model for self-efficacy.

In H3 and H4, this study predicted that SE mediates the relationship between PA and both LS and PR. Consequently, Model 4 of the PROCESS macro was used to analyze data from 560 participants. After adjusting for gender, grade level, and age, the results of the regression analysis are shown in Table 3 and Fig 2, PA significantly predicted SE (β = 0.398, *p* < 0.001), LS (β = 0.218, *p* < 0.001), and PR (β = 0.179, *p* < 0.001) among college students. SE significantly predicted LS (β = 0.489, *p* < 0.001) and PR (β = 0.805, p < 0.001). Therefore, the indirect effects of PA on LS and PR through SE were significant, with indirect effects of 0.195 and 0.320, respectively, and 95% CI of [0.138, 0.255] and [0.245, 0.399]. Thus, H3 and H4 are supported.

**Table 3:** Mediating effect of self-efficacy.

Outcome Variable	Predictor Variable	*β*	*SE*	*T*	Bootstrap 95% CI		*R²*	*F*
					Boot LLCI	Boot ULCI		
SE	PA	0.398***	0.041	9.758	0.318	0.478	0.210	36.779
LS	PA	0.218***	0.039	5.641	0.143	0.295	0.392	71.316
	SE	0.489***	0.037	13.115	0.416	0.562		
PR	PA	0.179***	0.022	7.982	0.135	0.223	0.797	435.211
	SE	0.805***	0.022	37.373	0.762	0.847		

Note: PA, physical activity; LS, life satisfaction; PR, psychological resilience; SE, self-efficacy; PS, perceived stress; ***p < 0.001.

#### 4.3.2. The mediating role of perceived stress.

**Fig 3:**
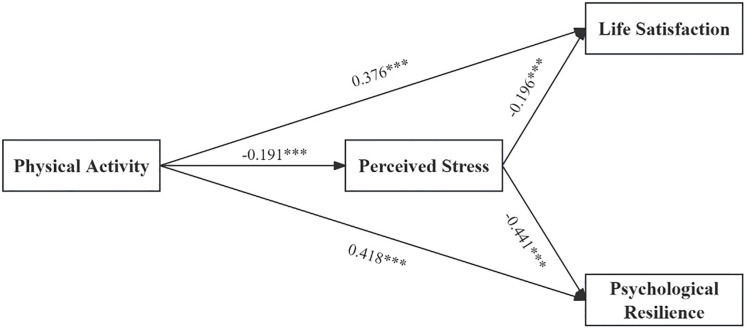
Results of the mediating effect model for perceived stress.

In H5 and H6, this study predicted that PS mediates the relationship between PA and both LS and PR. Accordingly, Model 4 of the PROCESS macro was used to analyze data from 560 participants. After adjusting for gender, grade level, and age, the results of the regression analysis are shown in Table 4 and Fig 3, PA significantly negatively predicted PS (β = −0.191, *p* < 0.001) and significantly positively predicted LS (β = 0.376, *p* < 0.001) and PR (β = 0.418, *p* < 0.001). PS significantly negatively predicted LS (β = −0.196, *p* < 0.001) and PR (β = −0.441, *p* < 0.001). Therefore, the indirect effects of PA on LS and PR through PS were significant, with indirect effects of 0.037 and 0.084, respectively, and 95% CI of [0.013, 0.070] and [0.040, 0.134]. Thus, H5 and H6 are supported.

**Table 4:** Mediating effect of perceived stress.

Outcome Variable	Predictor Variable	β	SE	T	Bootstrap 95% CI		*R²*	*F*
					Boot LLCI	Boot ULCI		
PS	PA	−0.191***	0.044	−4.344	−0.278	−0.105	0.081	12.178
LS	PA	0.376***	0.041	9.222	0.296	0.456	0.238	34.575
	PS	−0.196***	0.039	−5.053	−0.271	−0.120		
PR	PA	0.418***	0.034	12.132	0.348	0.482	0.464	95.941
	PS	−0.441***	0.032	13.587	−0.505	−0.377		

Note: PA, physical activity; LS, life satisfaction; PR, psychological resilience; SE, self-efficacy; PS, perceived stress; ***p < 0.001.

## 5. Discussion

This study aimed to thoroughly explore the comprehensive impacts of PA on LS and PR among college students, along with the mediating roles of SE and PS. Through a comprehensive review of existing literature, this study hypothesized that PA plays a significant role in enhancing LS and PR in college students, and that SE and PS significantly mediate these relationships. The findings supported these hypotheses, showing that PA significantly improved LS and PR among college students. Additionally, the SE and PS respectively mediated the relationships between PA and both LS and PR. More importantly, this study clearly delineates the complex mechanisms through which PA promotes positive psychological outcomes (LS and PR) in college students by examining two key mediating variables simultaneously. Specifically, it highlights how PA enhances SE and reduces PS through two parallel and interconnected pathways. This significantly advances previous research that primarily focused on direct effects or single mediators. The following section will discuss these findings in detail and explore their implications for deepening theoretical understanding and guiding practical applications.

### 5.1. Impact of physical activity on life satisfaction

PA has a significant positive impact on LS among college students, a finding consistent with the study by Liu, Zhu and Jiang [19], which found that appropriate PA significantly predicts higher LS in college students. Participation in PA provides opportunities for increased social interaction, fostering the formation of friendships and expansion of social support networks. Good social relationships enhance individuals’ sense of belonging and well-being, thereby improving LS [59]. According to SDT [21], the fulfillment of basic psychological needs such as relatedness, competence, and autonomy, which are often nurtured through physical activity, plays a crucial role in promoting well-being and LS [60]. When students engage in PA, they experience a sense of accomplishment and competence (especially in activities like team sports or improving personal fitness levels), which directly contributes to their sense of LS. Additionally, participating in team sports can cultivate team spirit and cooperative skills, which not only enhance individuals’ social skills but also bring positive psychological effects, further increasing LS [61]. Moreover, PA helps regulate emotions, reduce symptoms of depression and anxiety, and increase the frequency of positive emotions. A good emotional state is an important component of high LS [62]. This research finding further validates and strengthens the evidence supporting the direct positive effect of PA on LS, providing new support for this core relationship.

### 5.2. Impact of physical activity on psychological resilience

PA has a significant positive impact on the PR of college students, aligning with the findings of Zhao, Zhao, Wang, Zhang and Chen [11], who observed that college students who regularly engage in PA demonstrate higher emotional regulation capabilities and PR. PA improves physical health and enhances fitness, providing students with a solid physiological foundation when facing life’s challenges. SET emphasizes that physical fitness provides “physiological evidence” that supports the belief in one’s ability to cope with challenges. From a process-oriented perspective in resilience theory, resilience develops as individuals overcome the negative impacts of exposure to harmful and traumatic events, which results from the interaction between individuals and their environment [63]. Physical activity is often associated with challenges, self-improvement, and goal achievement. During exercise, individuals are required to socialize and continuously adapt to their surroundings, helping them acquire the skills and abilities to better navigate difficulties and challenges [64]. Furthermore, regular physical activity helps balance hormonal levels, such as reducing the secretion of stress hormones (cortisol) and increasing the production of happiness hormones (endorphins), thereby enhancing PR [12,13]. PA also serves as an effective emotional regulation tool, helping to reduce emotional fluctuations and manage negative emotions, thus enabling students to maintain mental toughness when facing academic and life challenges [65]. This research finding further consolidates the theoretical foundation of PA as a direct driving factor for enhancing PR in college students.

### 5.3. The mediating role of self-efficacy

SE plays a partial mediating role between PA and LS among college students. This finding aligns with the research by Tian, Zhou, Qiu and Zou [32], which observed that college students who participate in PA exhibit higher SE, further enhancing their LS. When college students achieve specific goals through PA, such as running longer distances, lifting heavier weights, or improving sports performance, they not only develop a belief in their capabilities within the realm of sports but also in other areas of life. According to SET [26], achieving success in specific activities strengthens an individual’s belief in their own competence. This belief in one’s capabilities is not limited to the context of physical activity but extends to other life domains, thereby increasing LS [33]. Therefore, the increase in SE due to PA provides a psychological foundation for enhancing students’ overall LS, as they feel more capable of managing challenges and achieving goals in various areas of life. The enhanced sense of SE also fosters a greater sense of control, which is essential for psychological well-being [34]. By incorporating SE as a mediating variable into a more comprehensive model, this study not only confirms the unique mediating role of SE in the “PA → LS” pathway, but also provides new and more systematic evidence for understanding how PA enhances LS by strengthening individuals’ efficacy beliefs.

SE also partially mediates the relationship between PA and PR in college students. This is consistent with the findings of Li, Wang, Yu, Liu, Xu, Lin, et al. [39], which showed that appropriate PA significantly enhances the PR of college students, with SE significantly mediating this relationship. Small victories in PA, such as completing a challenging training session or achieving a new personal record, provide students with direct experiences of success. These achievements reinforce their SE, the belief in their capacity to reach goals [61]. Enhanced SE gives students greater confidence when facing challenges and difficulties, fostering a belief in their ability to overcome these obstacles. This belief is central to PR, as it motivates individuals to persist and seek solutions in adversity. SE also helps individuals more effectively manage and regulate their emotions [66], enabling them to maintain a more stable and positive mindset when confronted with stress and adversity. This study clearly identifies and validates the contribution of SE as a mediating variable in the model exploring the relationship between PA and PR. This finding deepens our understanding of how PA builds PR by fostering the belief of “I can do it,” providing more focused theoretical support for intervention strategies aimed at enhancing SE.

### 5.4. The mediating role of perceived stress

PS partially mediates the relationship between PA and LS among college students. This finding is consistent with the research by Meyer, Grob and Gerber [42], which indicated that PA can reduce college students’ perception of stress, thereby increasing their LS. Continuous high levels of stress not only affect an individual’s mental health and quality of life but can also lead to emotional issues and physical symptoms. Regular PA helps improve emotional stability and reduce emotional fluctuations among college students. SDT further explains that autonomous exercise (such as voluntary fitness activities) reduces the “sense of control” and decreases the perception of threat in stress evaluations. The enhanced SE and improved physical sensations from exercise can increase individuals’ sense of control and satisfaction, effectively reducing their perception of stress [41]. Stress-Coping Theory explains this finding by suggesting that engaging in physical activity helps reduce physiological stress responses, which enables individuals to better manage daily stressors [67]. Lower stress levels facilitate better decision-making and promote a positive outlook on life, which, in turn, improves LS [61]. Furthermore, the reduction in PS enables college students to make more rational and effective decisions in their academic and personal lives, thus enhancing their overall satisfaction with life [43]. This research finding clearly confirms that PA enhances LS through an independent pathway by effectively alleviating the stress levels experienced by individuals. It highlights the key mechanism of stress management in enhancing subjective well-being and provides more direct empirical evidence for designing interventions that focus on improving LS through exercise-based stress reduction.

PS also partially mediates the relationship between PA and PR among college students. This supports the conclusions by Lines, Ducker, Ntoumanis, Thogersen-Ntoumani, Fletcher and Gucciardi [47], who found that college students actively involved in team sports exhibited lower levels of PS and enhanced their PR through this pathway. PA activates the body’s physiological response systems, prompting the brain to release natural chemicals like endorphins, which alleviate pain and enhance feelings of pleasure. Additionally, PA helps reduce the levels of stress hormones (such as cortisol) in the body, thereby reducing feelings of stress [12,13]. SDT posits that cooperation in team sports satisfies the “relatedness need,” which can reduce feelings of loneliness and further decrease PS. When college students engage in team sports through PA, it effectively reduces their PS, they often find themselves better equipped to handle life’s challenges. This experiential learning enhances their coping strategies and increases their resilience in stressful situations [46]. Moreover, consistent PA and sustained stress reduction help individuals develop a more positive outlook on life and worldview [68]. This positive mindset is helpful in maintaining optimism when facing difficulties, further enhancing PR. This result clearly reveals that the reduction of PS is an important mechanism through which PA enhances PR in college students. It significantly deepens our understanding of how PA acts as a “stress buffer” to strengthen individuals’ ability to cope with adversity, providing a more reliable foundation for developing programs aimed at lowering PS through exercise interventions to enhance resilience.

## 6. Implications and limitations

### 6.1. Implications

This study delved into the comprehensive effects of PA on LS and PR among college students, examining in detail the mediating roles of SE and PS. This research not only confirmed the positive impact of PA on mental health but also highlighted the critical roles of SE and PS in promoting mental health processes. Theoretically, this study expands the discussion on LS and PR, emphasizing the potential of PA as an effective intervention tool, and further enriches the research content of positive psychology and sports psychology. By exploring the mediating roles of SE and PS, this study offers new perspectives on how PA can specifically improve mental health. These findings provide a theoretical basis for universities to design and implement interventions aimed at enhancing students’ mental health and LS, and they underscore the importance and necessity of promoting PA among student populations.

Based on the core finding of this study that PA significantly enhances college students’ LS and PR, this paper provides specific practical recommendations for policymakers, universities, and students. Firstly, education policymakers should place greater emphasis on the vital role of PA in promoting college students’ physical and mental health, considering it a core strategy for improving overall student well-being. Policies should encourage and support investments in sports facilities and diversified physical activities (such as physical education courses, campus sports events, and social sports activities) to facilitate broader student participation in PA. Additionally, preferential policies can be implemented to encourage universities to offer personalized physical activity options tailored to different interests and needs, thereby increasing student engagement and motivation for exercise. Secondly, university administrators should fully recognize that PA not only contributes to students’ physical health but also indirectly enhances their LS and PR through mechanisms such as boosting SE and alleviating stress. Therefore, universities should actively invest in sports facilities, offer regular PA courses, organize diverse physical activities, and provide students with multi-layered participation opportunities by combining academic and extracurricular activities. Universities can also enhance students’ teamwork spirit and social skills by organizing sports competitions, team activities, and other events, which will further improve students’ mental health. Lastly, individual students should recognize that regular participation in PA is not only beneficial to their physical health but also significantly improves their LS and PR. Therefore, students should proactively choose physical activities that suit their interests and set clear exercise goals. By continuously achieving these goals, students can enhance their SE and stress management skills. Through participation in physical activities, students can not only relieve academic pressure but also strengthen their PR, enabling them to better cope with challenges in both their academic and personal lives, thus promoting the overall development of their physical and mental health.

### 6.2. Limitations and future research directions

While this study has advanced our understanding of the relationships among university students’ PA, LS, PR, SE, and PS, it also has several limitations. Firstly, this study utilized a cross-sectional design, collecting data at a single point in time, which does not establish causality. Although associations between PA and both LS and PR were found, it is unclear if PA directly leads to improvements in these psychological states. Future research could employ a longitudinal design, tracking the same subjects over different time points to explore the causal relationships between PA and both LS and PR. Secondly, this study primarily relies on self-reported data to assess the variables. Self-report data may be influenced by recall bias and social desirability effects, which limit the objectivity and accuracy of the data. Future research could improve the objectivity and reliability of the data by integrating multiple data collection methods, such as fitness tests and anthropometric measurements. Furthermore, the findings of this study provide valuable insights into the impact of PA on LS and PR among college students, but their generalizability may be limited. Cultural differences, educational disparities, and socio-economic factors across regions could affect how physical education influences students’ well-being. In some cultures, PA may be more emphasized, while others may face challenges such as limited resources or negative societal attitudes toward physical activity. Future research should explore cross-cultural differences and consider socio-economic and institutional factors to better understand how PA programs affect student well-being in diverse contexts. Finally, although this study considered the mediating roles of SE and PS, it was unable to fully control for other potential variables that may influence the results, such as personality traits (e.g., extraversion, neuroticism), family upbringing, peer support, family background, socioeconomic status, and academic pressure. Therefore, future research should take into account and control for additional variables that may affect the outcomes, such as family environment, social support, economic conditions, and academic stress, in order to improve the accuracy of the research conclusions.

## 7. Conclusion

This study found that PA was significantly positively associated with college students’ LS and PR. Furthermore, SE and PS played significant mediating roles in the relationship between PA and both LS and PR. Theoretically, this study enriches the model of the relationship between PA, LS, and PR, providing new perspectives for understanding the mechanisms through which PA is associated with college students’ mental health (i.e., via SE- and PS-related mediating pathways). Practically, this study offers theoretical support for the development of mental health intervention strategies for college students, particularly in leveraging the associations with enhanced SE and reduced PS. The limitations of this study include the use of a cross-sectional design, which limits causal inference; the narrow sample scope, which may affect the generalizability of the results; and the potential for subjective bias due to the use of self-report questionnaires. Future research could adopt a longitudinal design, expand the sample size, and combine multiple data sources to further validate and extend the model proposed in this study.
